# Association of smoking with the survival of patients with brain metastasis of lung cancer

**DOI:** 10.3389/fneur.2023.1036387

**Published:** 2023-03-13

**Authors:** Jiayi Yu, Yu Zhang, Zheran Liu, Yan He, Yiyan Pei, Renjie Zhang, Xingchen Peng, Fang Fang

**Affiliations:** ^1^School of Medical and Life Sciences, Chengdu University of Traditional Chinese Medicine, Chengdu, Sichuan, China; ^2^Department of Biotherapy, Cancer Center, West China Hospital, Sichuan University, Chengdu, China; ^3^Department of Neurosurgery, Affiliated Hospital of Chengdu University, Chengdu, Sichuan, China; ^4^Department of Neurosurgery, West China Hospital, Sichuan University, Chengdu, Sichuan, China; ^5^West China Hospital, Sichuan University, Chengdu, Sichuan, China

**Keywords:** smoking, lung cancer, brain metastasis, smoking cessation, the overall survival

## Abstract

**Background:**

Smoking is associated with increased mortality in patients with cancer. However, there are limited data on the impact of smoking on the survival of patients with brain metastases. Therefore, this study aimed to evaluate whether smoking was associated with survival and whether smoking cessation was beneficial to these patients.

**Methods:**

This study used lung cancer with a brain metastasis cohort of the West China Hospital of Sichuan University from 2013 to 2021. Patients were stratified according to smoking history; the distribution, clinical characteristics, and survival data of each group were estimated. Kaplan-Meier analysis and risk analysis were performed for the survival endpoint.

**Results:**

Of the 2,647 patients included in the analysis, the median age was 57.8 years, and 55.4% were men. Among them, 67.1% had no smoking history, 18.9% still smoked, and 14% reported quitting smoking. Compared with never smokers, current smokers [HR, 1.51 (95% CI, 1.35-1.69), *p* < 0.01] and former smokers [HR, 1.32 (95% CI, 1.16-1.49), *p*<0.01] had an increased risk of death. However, quitting smoking was not associated with improved survival [HR, 0.90 (95% CI, 0.77-1.04), *p* = 0.16]. The overall survival increased with the increase of smoking cessation years.

**Conclusions:**

In lung cancer patients with brain metastases, smoking was associated with an increased risk of death, but quitting smoking was not associated with improved survival.

## Introduction

Lung cancer is the leading cause of cancer-related death (1). Approximately 81.3% of patients with lung cancer are related to smoking (2). Although the overall smoking prevalence has decreased significantly in the past years, cigarette smoking remains the leading cause of lung cancer cases and deaths (3). Smoking in patients with cancer increases overall mortality, cancer-specific mortality, treatment-related toxicity, and second primary cancer (4, 5). The 2020 Surgeon General's Report on quitting smoking added many new pieces of evidence and conclusions to reveal the benefits of quitting smoking even after the diagnosis of cancer (6). The American Association for Cancer Research, EMSO, the WHO, and other organizations advocate smoking cessation as a standard cancer treatment (7–10). More than half of lung cancers develop into brain metastasis during the course of the disease (11). However, guidelines rarely specifically recommend smoking cessation for brain metastasis. Brain metastasis is a sign of poor prognosis in patients with lung cancer, and the expected survival time is short (12). Patients with advanced cancer are willing to quit smoking differently from those diagnosed early. Pain, second-hand smoke exposure, guilt about smoking, fear of stigmatization, and fatalism of disease may represent obstacles to smoking cessation in patients with cancer, particularly in those with advanced disease (13–16).

There is less evidence about the impact of smoking on the survival of patients with brain metastases and the benefits of quitting smoking on patients with brain metastases. Only a small study of 366 brain metastases in patients with lung cancer found that smoking had no effect on overall survival (17). Therefore, we conducted this study to evaluate whether smoking affects the overall survival rate of lung cancer patients with brain metastasis and the benefits of quitting smoking on survival.

## Methods

### Study population

This study is a single-center retrospective cohort study on the survival rate of lung cancer patients with brain metastasis in China. From December 2013 to August 2021, lung cancer patients with brain metastasis coded C34 and secondary invasive C79.3 were screened from the West China Hospital of Sichuan University database according to the 10th edition of the International Classification of Diseases for Oncology (18).

### Data collection and follow-up

Electronic medical records and social population registration records collected information about demographic characteristics, family, medical history, and survival time. We actively collected data on smoking behavior through the electronic medical record, including the years of smoking, the average amount of smoking per day, whether they had quit smoking, and the time they had quit smoking. We multiplied the number of cigarette packs smoked per day by the patient's number of years and calculated the cumulative smoking per pack-year (based on 20 cigarettes per pack). A patient was classified as a current smoker if he/she had evidence of active smoking or had < 1 year of smoking cessation. Moreover, we classified patients who quit smoking at least 1 year before diagnosis as former smokers.

Patients' general conditions when diagnosing diseases were also obtained, including anthropometric data, functional status, chronic health conditions, and Karnofsky performance status (KPS) score. The number of brain metastases and other metastases was also recorded. We also collected their history of drinking in their life. Drinking is defined as drinking alcoholic drinks at least one time a week in a year. We sorted all relevant treatment history, imaging, and histopathological information into the patient's data, and determined electronic medical records and follow-up records after the patient's diagnosis, life state, tumor progression, and the treatment process during the disease. This study was approved by the ethics committees of the West China Hospital and the written consent for patients included 161 in the study was exempted by the ethics committees since the study only used retrospective observation data (No. 2022127).

### Statistical analyses

Descriptive statistics were conducted to summarize baseline characteristics, with numbers and percentages for categorical variables and means for continuous variables. We imputed the missing values with the average value. Hazard ratios (HR) and 95% confidence interval (95% CI) for survival associated with smoke were estimated using Cox proportional hazards model. Stratified analyses and multivariate Cox proportional hazards analyses controlled for potential confounding. The multivariable model was adjusted according to the age, gender, KPS score at diagnosis, histological type (including adenocarcinoma and non-adenocarcinoma), past medical history (including diabetes mellitus and hypertension), the number of intracranial metastases, the presence of extracranial metastasis, the radiotherapy alone, target therapy alone, chemotherapy alone, the cumulative amount of smoking at diagnosis, whether to quit smoking, the time of quitting smoking, and the drinking status at diagnosis. The model covariates were selected based on the available literature to include the suspected prognostic factors for lung cancer with brain metastasis survival and the variables that might influence the assessed exposure (19–22). Kaplan–Meier survival curves were used to compare the survival of current, former, and never smokers, and the log-rank test was used to test this difference.

In this model, the start time is defined as the patient's first admission date and diagnosis as brain metastasis of lung cancer. To assess the effect of smoking and quitting smoking on overall survival, the date of death from any cause was set as the end time. If the patient is still alive within the follow-up time, we set the follow-up time (15 August 2021) as the end date for the patient. Overall survival (OS) was defined as the interval from diagnosis of brain metastases to death. A subgroup analysis was also performed among current and former smokers, including the smoking intensity and duration since smoking cessation. *P*-values that were reported as two-sided and < 0.05 were considered statistical differences. All statistical analyses were bilateral and performed using R statistical software (version 4.0.3, Vienna, Austria).

## Results

The study collected 2,647 lung cancer patients with brain metastasis, the median age was 57.8, and 55.4% were men. Among them, 67.1% had no smoking history, 18.9% still smoked, and 14% reported quitting smoking. The median overall survival for the cohort was 2 years (95%CI: 1.9–2.1). The median overall survival rates of never smokers, former smokers, and current smokers were 2.2 years (95%CI: 2.1–2.3), 1.7 years (95%CI: 1.5–1.9), and 1.5 years (95%CI: 1.3–1.7), respectively. The baseline characteristics of patients are shown in Table 1.

**Table 1:** Clinical and smoking characteristics of lung cancer patients with brain metastases.

**Characteristic**	**All patients**	**Smoke**			***P*-value**
		**Never**	**Former**	**Current**	
Participants, *n* (%)	2,647 (100.0)	1,775 (67.1)	371 (14.0)	501 (18.9)	
Mean age (SD), y	57.8 (11.0)	57.4 (11.5)	59.6 (10.3)	57.9 (9.8)	0.002
Gender, *n* (%)					< 0.001
Male	1,468 (55.4)	620 (35.0)	362 (97.5)	486 (97.0)	
Female	1,179 (44.6)	1,155 (65.0)	9 (2.5)	15 (3.0)	
Histology, *n* (%)					< 0.001
Adenocarcinoma	2,416 (91.3)	1,672 (94.1)	316 (85.1)	428 (85.4)	
Non-adenocarcinoma	231 (8.7)	103 (5.9)	55 (14.9)	73 (14.6)	
KPS, *n* (%)					0.593
≤ 70	636 (24.0)	424 (23.8)	84 (22.6)	128 (25.5)	
>70	2,011 (76.0)	1,351 (76.2)	287 (77.4)	373 (74.5)	
Past medical history, *n* (%)					
Hypertension	439 (16.5)	287 (16.1)	72 (19.4)	80 (15.9)	0.287
Diabetes	233 (8.8)	126 (7.0)	53 (14.2)	54 (10.7)	< 0.001
Alcohol drinking, *n* (%)					< 0.001
Yes	545 (20.6)	69 (3.8)	188 (50.9)	288 (57.4)	
No	2,102 (79.4)	1,706 (96.2)	183 (49.1)	213 (42.6)	
BMI, *n* (%)					0.005
< 18.5	221 (8.4)	157 (8.8)	20 (5.4)	44 (8.8)	
18.5–23.9	1,801 (68.0)	1,228 (72.6)	239 (64.4)	334 (66.7)	
≥24	625 (23.6)	390 (18.6)	112 (30.2)	123 (24.5)	
Number of brain metastases, *n* (%)					0.677
< 3	1,680 (63.4)	1,128 (63.5)	241 (64.9)	311 (62.0)	
≥3	967 (36.6)	647 (36.5)	130 (35.1)	190 (38.0)	
Extracranial metastasis, *n* (%)					0.006
Yes	515 (19.4)	321 (18.1)	94 (25.3)	100 (19.9)	
No	2,132 (80.6)	1,454 (81.9)	277 (74.7)	401 (80.1)	
Treatment, *n* (%)					
Chemotherapy alone	445 (16.8)	282 (15.8)	57 (15.3)	106 (21.1)	0.015
Radiotherapy alone	80 (3.0)	49 (2.7)	16 (4.3)	15 (2.9)	0.283
Target therapy alone	147 (5.5)	116 (6.5)	9 (2.4)	22 (4.3)	0.003

BMI, body mass index (recorded when brain metastases was diagnosed); KPS, Karnofsky performance status.

**Figure 1:**
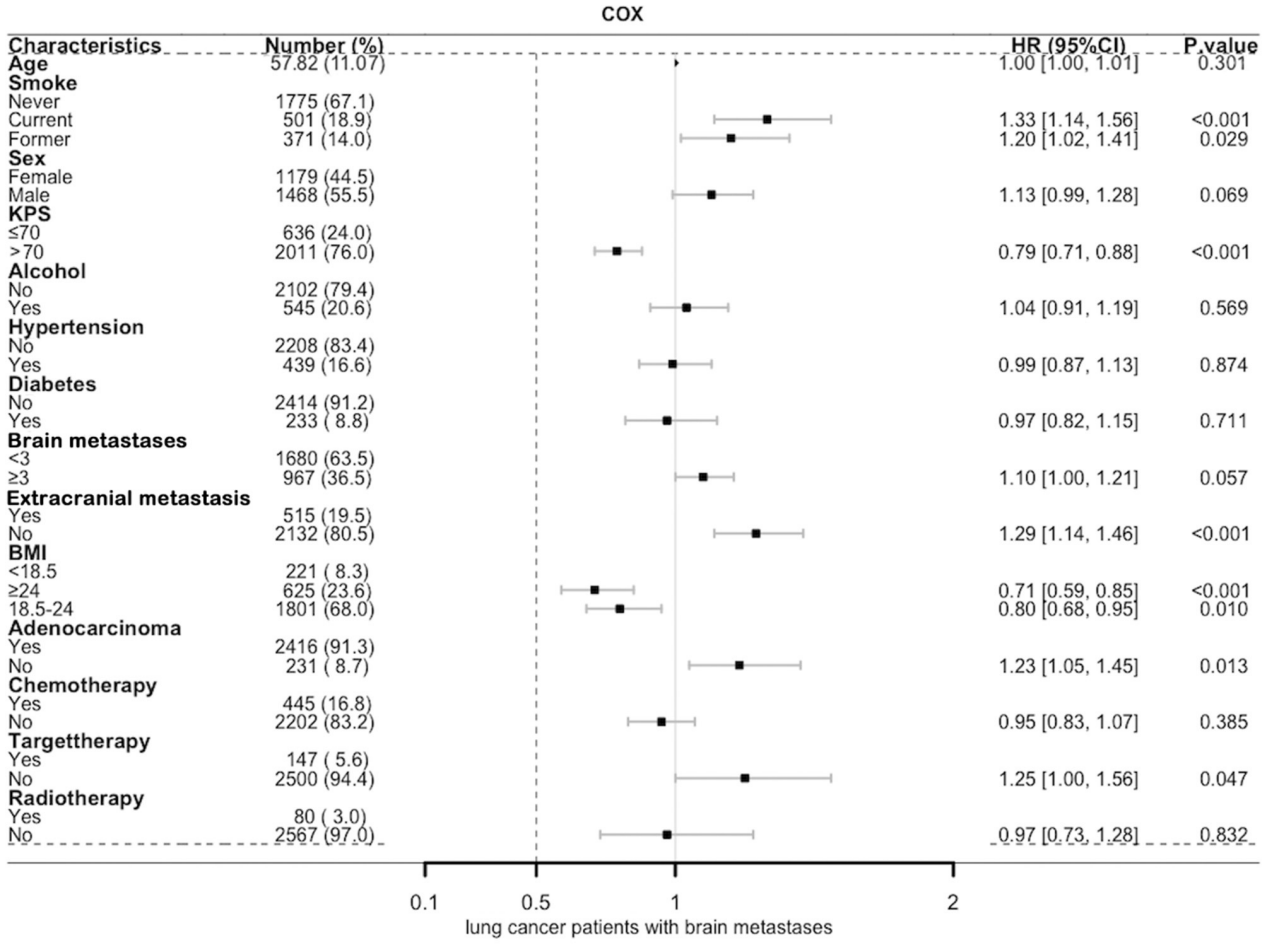
Multivariate Cox forest map of lung cancer patients with brain metastases. BMI, body mass index (recorded when brain metastases was diagnosed); KPS, Karnofsky performance status; Brain metastases, number of brain metastases.

**Figure 2:**
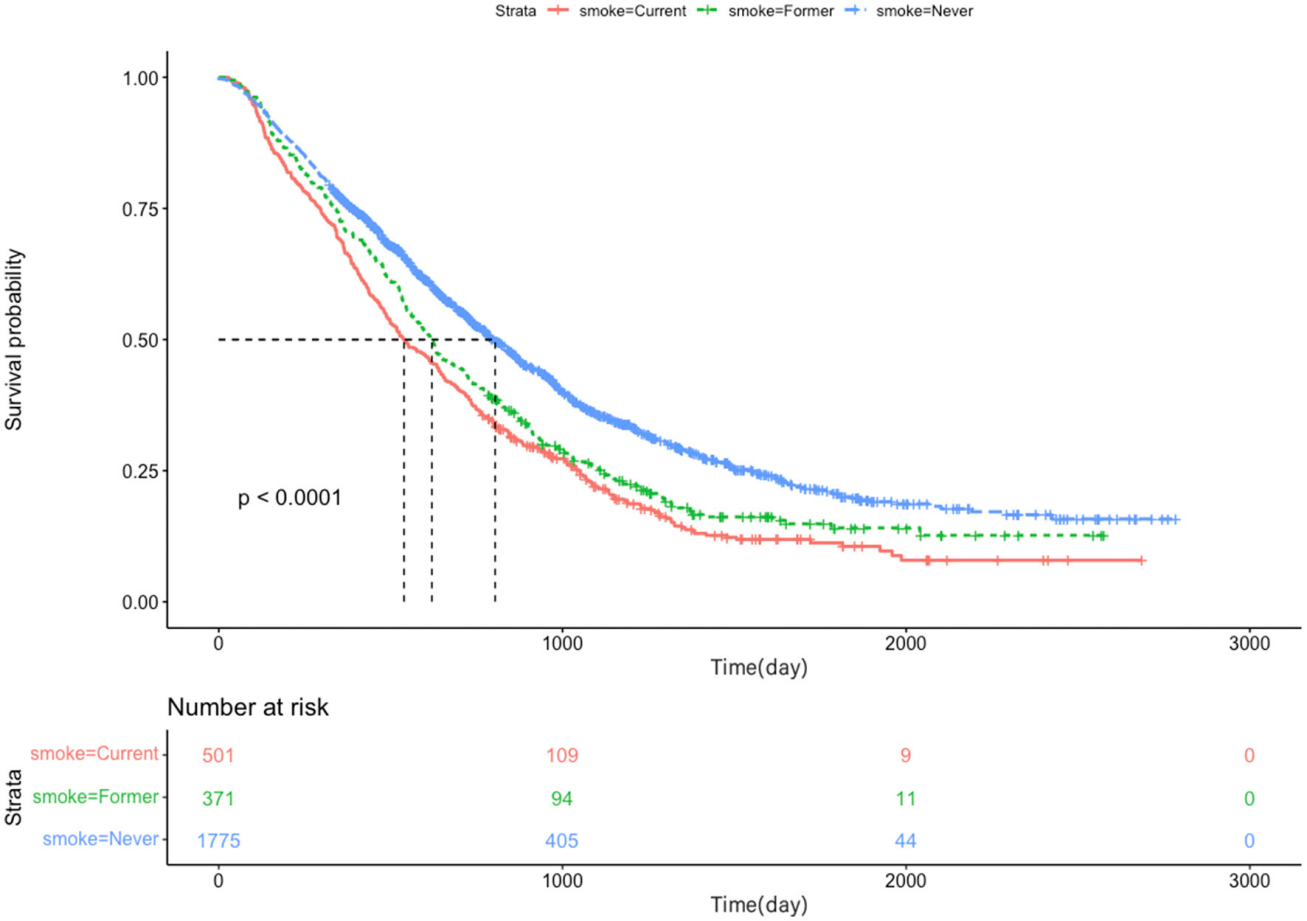
Kaplan–Meier plots of overall survival for lung cancer patients with brain metastases based on smoking status.

**Figure 3:**
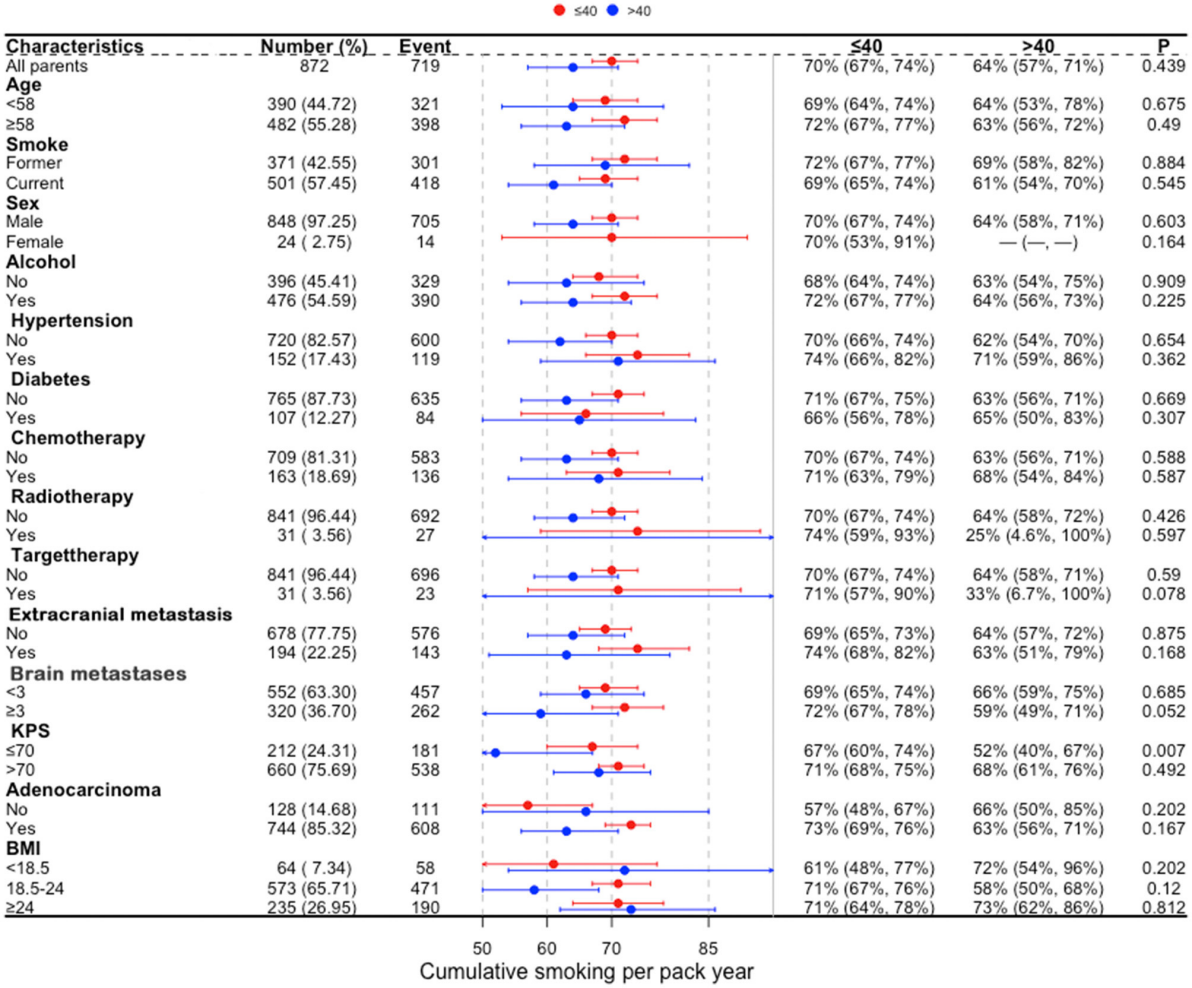
Subgroup analysis of cumulative smoking in current smokers and former smokers for lung cancer patients with brain metastases. BMI, body mass index (recorded when brain metastases was diagnosed); KPS, Karnofsky performance status; Brain metastases, number of brain metastases.

**Figure 4:**
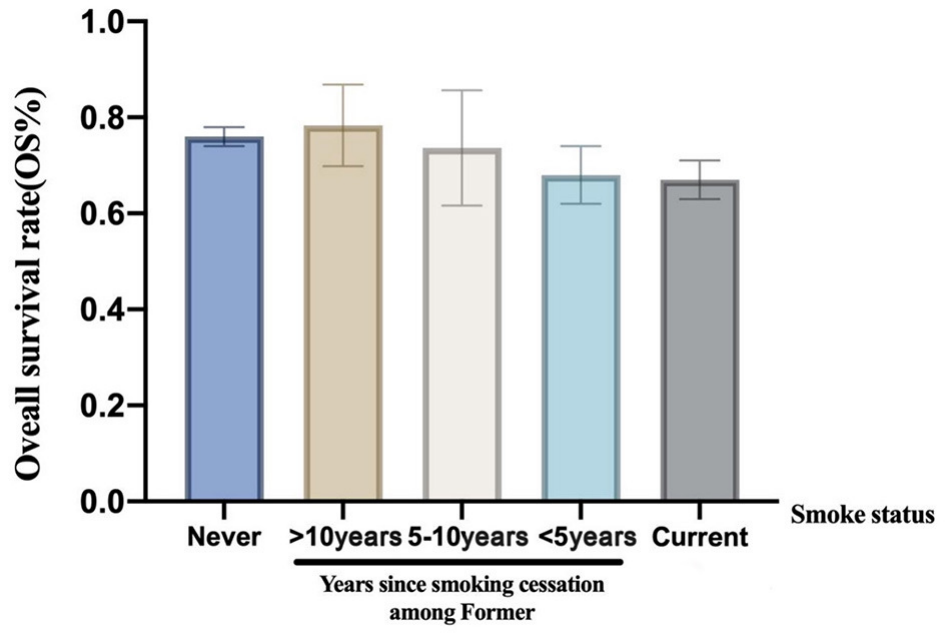
The overall survival rate stratified according to smoke status and years since tobacco cessation.

In a univariate analysis of the entire population, current smokers [HR, 1.51 (CI, 1.35–1.69), *p* < 0.01] and former smokers [HR, 1.32 (CI, 1.16–1.49), *p* < 0.01] had an increased risk of death compared with never smokers. After adjustment for potential confounders and risk factors, smokers (current and former smokers) still had an increased risk of death (Figure 1). However, compared with the current smoker, we did not see the benefits of quitting smoking for the overall survival of patients [HR, 0.90 (CI, 0.77–1.04), *p* = 0.16] (Supplementary Figure 1). Figure 2 shows the Kaplan–Meier plots of overall survival for patients based on smoking status. The cumulative pack years of smoking and smoking cessation duration of 371 former smokers and 501 current smokers were analyzed by subgroup analysis. According to the Cox proportional hazard analysis, annual cumulative smoking was used as a categorical variable, and smokers were divided into cumulative package years of ≤ 40 pack-year and cumulative package years of >40 pack-year; we found that cumulative smoking was not associated with overall survival in current smokers and former smokers (Figure 3). To assess the “time response” effect of quitting smoking, a total of five groups were generated according to the time of quitting smoking: current smokers, quitting smoking for 1–5 years, quitting smoking for 5–10 years, quitting smoking for ≥10 years, and never smokers. Figure 4 shows that the overall survival rate increased with smoking cessation years, but this was not statistically significant.

## Discussion

This retrospective study analyzed the survival of 2,647 lung cancer patients with brain metastasis. The results showed that smoking affected the overall survival rate of lung cancer patients with brain metastasis. However, smoking cessation was not associated with higher survival than current smoking.

Contrary to our study, previous studies did not find an association between smoking and lung cancer brain metastasis. Kim et al. analyzed 313 non-small-cell lung cancer (NSCLC) patients with brain metastasis, and univariate analysis showed that smoking affected the overall survival; however, there was no difference in the overall survival in the multivariate analysis (23). In another study of patients with non-small-cell lung cancer, only non-smokers in stage I had a significant survival advantage over smokers, and smokers who quit smoking in stage II or III disease had no significant reduction in the risk of death (24). But our study found that smoking increased the risk of death in lung cancer patients with brain metastases. This difference in part may be explained by our large size simple to make the results more precise.

Previous studies have shown that smoking increases the risk of brain metastasis (17, 25). Nicotine promotes brain metastasis by polarizing microglia and inhibiting innate immune function (25, 26). In addition, smoking affects the effect of chemotherapy in advanced patients (27), and a significant difference in tumor biology is the higher EGFR mutation rate of never smokers, which may explain the better prognosis of never smokers after treatment for the EGFR gene (28). It is shown that smoking and tobacco products alter biological pathways of cancer leading to increased proliferation, invasion, migration, angiogenesis, decreased response to cytotoxic therapy, and activation of pro-survival cellular pathways (29, 30). These may be the reasons for the difference in prognosis between never smokers and patients with a history of smoking. Some study also suggests that there is a dose-dependent relationship between smoking and the survival of patients with lung cancer (31, 32). In contrast to these results, our study showed that compared with light to moderate smokers, the overall survival of the heavy smoker (>40 pack-years) was not significantly decreased.

Smoking is a main prognostic factor of lung cancer. Evidence has shown that smokers who quit smoking for more than 1 year had higher survival than current smokers. Zhou et al. suggested that overall survival increased with the increase in smoking cessation time among patients with early-stage NSCLC (33). Nia et al. concluded that patients with early-stage NSCLC who quit smoking have significantly less mortality than current smokers (34). In a study of 4,200 smokers in the National Comprehensive Cancer Network NSCLC cohort, only young patients with stage IV disease who quit smoking >12 months before the diagnosis gained survival benefits (35). However, we did not find the benefits of quitting smoking for lung cancer patients with brain metastasis. Our study suggested that long-term continuous quitting smoking may have the trend of increasing survival. Paradoxically, when patients with lung cancer have brain metastasis, the median survival time is short, thus, they may not get the benefits of long-term quitting smoking. However, cumulative smoking cessation time before the diagnosis of brain metastasis may be essential to improve survival.

This study has several limitations. First, the patient's smoking history and smoking cessation are from electronic medical records, which will deviate from the patient's self-report. A study revealed that up to 50% of cancer patients' self-reporting about smoking may be inaccurate (36). Second, our information collection on tobacco intake is based on cigarettes, ignoring alternative products such as tobacco and nicotine. Third, although the large sample size is a strength of our study, this is a study of a single agency and the patients included in the cohort are all Asian. Therefore, the generalizability of the results to other populations is questionable.

## Conclusion

Different from previous studies, this study is not limited to patients with non-metastatic lung cancer. Although the survival time of patients with metastatic lung cancer is short, our results still show the harm of smoking to patients with brain metastasis of lung cancer. The survival rate of patients with smoking is lower than that of patients without smoking. We failed to find the relationship between quitting smoking and survival.

## Data availability statement

The raw data supporting the conclusions of this article will be made available by the authors, without undue reservation.

## Ethics statement

Ethical review and approval was not required for the study on human participants in accordance with the local legislation and institutional requirements. Written informed consent from the patients/participants or patients/participants' legal guardian/next of kin was not required to participate in this study in accordance with the national legislation and the institutional requirements.

## Author contributions

XP and FF conceived the study. JY performed the main analysis and wrote the original draft. ZL retrieved the data. YZ participates in the revision of the manuscript. The study reported in the manuscript has been performed by JY, YZ, ZL, YH, YP, RZ, XP, and FF unless clearly specified in the text. All authors read, approved the manuscript, and conducted data screening and collation.

## Supplementary material

Supplementary Figure 1 Multivariate Cox forest map of lung cancer patients with brain metastases. BMI, body mass index (recorded when brain metastases was diagnosed); KPS, Karnofsky performance status; Brain metastases, number of brain metastases.
