# Supplementary figures and images for: Association of smoking with the survival of patients with brain metastasis of lung cancer
Source: Front Neurol. 2023 Mar 13;14:1036387. doi: 10.3389/fneur.2023.1036387 (PMC10040669; doi:10.3389/fneur.2023.1036387)

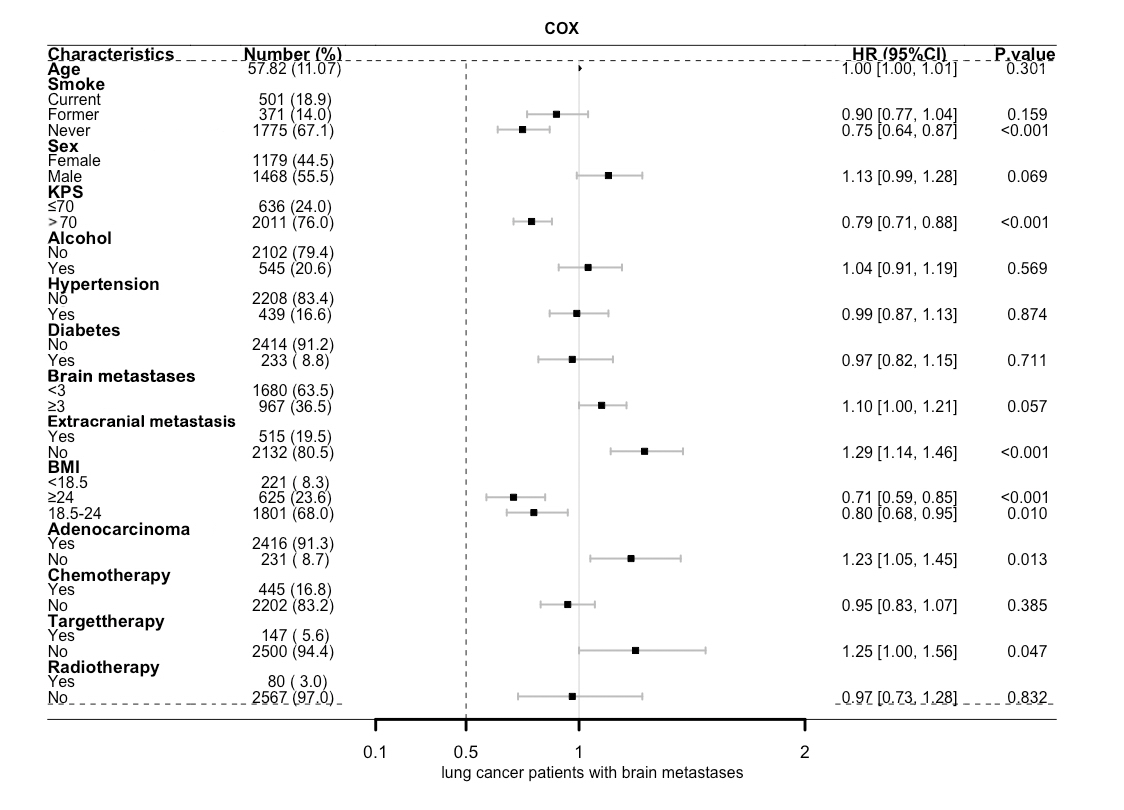

Supplement: Supplementary Figure 1 — Multivariate Cox forest map of lung cancer patients with brain metastases. BMI, body mass index (recorded when brain metastases was diagnosed); KPS, Karnofsky performance status; Brain metastases, number of brain metastases. [file Image_1.TIF]
